# Audio object classification using distributed beliefs and attention

**DOI:** 10.1109/taslp.2020.2966867

**Published:** 2020-01-15

**Authors:** Ashwin Bellur, Mounya Elhilali

**Affiliations:** Department of Electrical and Computer Engineering, Laboratory for Computational Audio Perception, Johns Hopkins University.

**Keywords:** Deep belief network, Distributed processing, Attention, Acoustic objects, Robust classification

## Abstract

One of the unique characteristics of human hearing is its ability to recognize acoustic objects even in presence of severe noise and distortions. In this work, we explore two mechanisms underlying this ability: 1) redundant mapping of acoustic waveforms along distributed latent representations and 2) adaptive feedback based on prior knowledge to selectively attend to targets of interest. We propose a bio-mimetic account of acoustic object classification by developing a novel distributed deep belief network validated for the task of robust acoustic object classification using the UrbanSound database. The proposed distributed belief network (DBN) encompasses an array of independent sub-networks trained generatively to capture different abstractions of natural sounds. A supervised classifier then performs a readout of this distributed mapping. The overall architecture not only matches the state of the art system for acoustic object classification but leads to significant improvement over the baseline in mismatched noisy conditions (31.4% relative improvement in 0dB conditions). Furthermore, we incorporate mechanisms of attentional feedback that allows the DBN to deploy local memories of sounds targets estimated at multiple views to bias network activation when attending to a particular object. This adaptive feedback results in further improvement of object classification in unseen noise conditions (relative improvement of 54% over the baseline in 0dB conditions).

## I. Introduction

The ability of the human brain to make sense of complex acoustic information in everyday scenes exploits intricate transformations along a hierarchical biological network that maps low dimensional acoustic signals into rich high-dimensional representations. Studies of the auditory system have shed light on the span and complexity of these transformations and showed that the signal entering our ears is mapped onto increasingly compound spaces that encode detailed spectral, temporal and spatial dynamics [1–5]. These transformations can be viewed as mappings of the signal onto a high dimensional feature space that spans spectrotemporal modulations of natural sounds and allows the interpretation of acoustic signals into perceptual objects [6, 7]. Recent work based on functional magnetic resonance imaging (fMRI) suggests that this encoding happens through forming *multiple* views of the time-frequency spectrogram with varying degrees of spectrotemporal resolutions [8, 9]. These results suggest the existence of a complex spatially distributed neural network in cortical regions that forms a scattered representation of the spectrotemporal characteristics of a complex sound, with each region capturing the scene from a particular vantage point. Cortical neurons in these regions essentially act as filters exhibiting selectivity to a particular section of the modulation profile of natural sounds. While the multiple view distributed representation can be redundant, it is hypothesized that it enables segregation of acoustic objects and also robust behavior by discriminatively highlighting distinct characteristics of sounds of interest and distractors that should be ignored.

Complementing this intricate sensory encoding are feedback mechanisms from cognitive brain networks that engage prior knowledge — in the form of memory — to guide our attention to the target sound. This attentional selection plays a crucial role in the robust behavior of brain networks when dealing with complex and ever-changing acoustic soundscapes and guides neural resources to process relevant information in the signal [6, 10–13]. Directing our attention to sounds of interest relies on an intricate circuitry that engages memory of known objects and deploys prior knowledge to modulate how incoming sounds are processed in order to maximize detectability of instantiations of these target objects. These mechanisms play a significant role in rendering the auditory system effective in dealing with complex and ever changing listening conditions in everyday environments [6, 10–13]. The representation of these memory constructs also likely operates in a distributed fashion rather than a unitary system [14–16]. These representations can then be deployed with various abstractions depending on which resolution is most suitable for the task at hand. Guidance from this local memory ultimately reshapes processing of the incoming sensory signal and provides the biological system with notable robustness and flexibility in dealing with unexpected distortions or changes in the environment [17].

In this work, we leverage this distributed processing of sensory information and local memory to explore benefits for a task of acoustic object classification. We propose a generative deep belief based framework to perform the sensory mapping from the time-frequency representation to the spectrotemporal modulation space. Expanding on the concept of convolutional restricted boltzmann machine (CRBM) [18], we propose a novel architecture referred to as a distributed belief network (DBN), to capture *multiple views* of the time-frequency representation of audio signals at different spectrotemporal resolutions. The DBN extends the standard single multi-layer hierarchical setup, into multiple local sub-networks (LSNs) organized in a hierarchical structure to propagate different temporal pooling ratios. The redundant encoding afforded by this network facilitates robust acoustic object representation. This claim is validated in a classification task by augmenting the DBN network with bi-directional long short-term memory (BLSTM) networks tested on classification of environmental sounds of the UrbanSound database [19]. The premise of distributed representation is further extended using local memories that inform attentional feedback to different sounds of interest. We introduce the concept of distributed local memory, where at each of the local sub-networks of the DBN we store a local memory of the acoustic object. In a task-driven setting, we develop mechanisms wherein the local memory is employed to induce attention at each of the local sub-networks during inference, thereby modulating the information encoded by the DBN as a whole, in a manner that enhances the acoustic object of interest. We show that incorporating such attentional mechanisms improves the robustness of the object classification system in presence of unseen noise distortions.

Recent works in machine vision and hearing have in fact leveraged the concept multiple levels of abstraction for inference and attention, more often implicitly, using deep neural networks. In [20], U-net was introduced, where the outputs from lower layers of the convolutional neural network (CNN) were also used during inference by *skipping connections* for biomedical image segmentation. Variations of such architecture were also found to be useful for tasks such as image to image translation [21]. Similar architectures based on skipping connections and exploring features at various levels of abstraction have been employed for tasks like singing voice separation [22] and music source separation [23]. The idea of attention has also gained prominence in the deep learning literature across applications such as document classification [24], image captioning [25], speech enhancement [26] and audio classification [27, 28]. Across this body of work, attention is also incorporated within the neural network framework though it is trained in an end-to-end manner.

In contrast, the present work adopts a generative distributed belief network to integrate and build on these ideas. The use of a generative inference enables us to explicitly train a feed-forward process in a task agnostic manner, hence allowing the exploration of tiling afforded by the distributed network to capture the spectrotemporal modulation space occupied by a large variety of naturally occurring sounds. The use of CRBMs as the basic building blocks enables us to approximate the cortical processes, allowing us to study the tuning characteristics of the proposed distributed belief network in relation to the distributed sensory processing observed in the mammalian auditory system [8, 9]. Using a feed forward belief network as a fixed feature extractor, also affords us the flexibility to probe the advantages of the redundant views captured by each of the sub-networks of the DBN and the DBN as a whole in mismatched settings. Further, attention is explored as a standalone process that can serve as an information bottleneck to modulates the features captured by the generative process. This enables us to study the role of attentional mechanisms in enhancing performance, particularly in terms of its manifestations in the spectrotemporal modulation space. Such detailed exploration of these processes at various levels of the network would be intractable in an end-to-end task-specific supervised system.

The outline of the paper is as follows: Section II describes the core convolutional deep belief network proposed in this study, while section III complements this representational network with supervised training to perform acoustic object classification using DBN mappings. Section IV extends the framework to explore ideas of bio-mimetic local memory and attentional feedback. Details of the experiments and results in section V and a discussion of the performance and lessons learned is presented in section VI.

## II. Distributed belief network

The sensory mapping process modeled in this work follows the hierarchical transformations that take place along the auditory system [1–3]. These transformations start at peripheral and mid-brain regions where the time domain waveform is transformed into a time frequency representation. In this work, we model these early mappings using the mathematical approximation proposed in [29], resulting in a time-frequency auditory spectrogram. Unlike a classic short-term Fourier transform, this spectrogram employs a log-scale asymmetric filterbank and includes nonlinear compression and high-pass and low-pass operations to mimic temporal resolutions observed in the biological system (see Chi et al. [29] for details).

The next stages in the hierarchy, particularly in auditory cortical regions, analyze details in the spectrotemporal profile of incoming signals [9]. The novel approach proposed in this work derives an array of spectrotemporal filters in a data driven manner by training unsupervised deep belief networks using convolutional restricted boltzmann machine (CRBM). This type of generative belief network for audio applications was first proposed in [18]. It employed linear spectrograms and convolution along the time dimensions, and showed that resulting bases are informative spectrotemporal patches about the incoming sounds. In the current work, we extend this approach to a distributed array of belief networks, which leverages the statistical characteristics of the data from different vantage points, as well as encode the spectrotemporal modulation space along increasingly abstract representations along a hierarchy. Before detailing the distributed belief network architecture, we briefly review the mathematical formulation of the CRBM setup (see [18] for details).

For the sake of simplicity, we consider the input layer to be a single channel of an auditory spectrogram. The formulation can be easily extended to a multi-channel setting. Let *v* be the input spanning *n*_*i*_ time frames, which is mapped via *n*_*b*_ bases of size 1 *× n*_*w*_. The hidden layer with units denoted as *h* has dimensions *n*_*b*_ *× n*_*p*_ where *n*_*p*_ = *n*_*i*_ − *n*_*w*_ + 1. For Bernoulli visible units, the energy function is defined as

(1) $$E(v,h)=-\sum_{b=1}^{n_{b}}\sum_{p=1}^{n_{p}}\sum_{r=1}^{n_{w}}h_{p}^{b}B_{r}^{b}v_{p+r-1}\;-\sum_{b=1}^{n_{b}}d_{b}\sum_{p=1}^{n_{p}}h_{p}^{b}-c\sum_{i=1}^{n_{i}}v_{i}$$

where *B* represents the *n*_*b*_ basis of size *n*_*w*_, $B_{r}^{b}$ being the *r*^*th*^ dimension of the *b*^*th*^ basis , *d*_*b*_ is the shared bias of the *b*^*th*^ basis and *c* is the shared bias for the visible units. For real-valued visible units, equation (1) is adapted as follows:

(2) $$E(v,h)=-\frac{1}{2}\sum_{i=1}^{n_{i}}v_{i}^{2}-\sum_{b=1}^{n_{b}}\sum_{p=1}^{n_{p}}\sum_{r=1}^{n_{w}}h_{p}^{b}B_{r}^{b}v_{p+r-1}\;-\sum_{b=1}^{n_{b}}d_{b}\sum_{p=1}^{n_{p}}h_{p}^{b}-c\sum_{i=1}^{n_{v}}v_{i}$$

The joint probability of visible and hidden units is then derived from this energy function and defined as:

(3) $$P(v,h)=\frac{1}{Z}exp(-E(v,h))$$

where Z is the partition function.

Condition probability of hidden units is defined as:

(4) $$P\left(h_{p}^{b}=1|v\right)=\text{sigmoid}\left({\left(B_{n_{w}-p+1}^{b}*v\right)}_{p}+d_{b}\right)$$

where * denotes convolution.

The conditional probability of visible units takes different forms depending on the nature of the visible units. For Bernoulli visible units, this conditional probability is defined as:

(5) $$P\left(v_{i}=1|h\right)=\text{sigmoid}\left(\sum_{b}{\left(B^{b}*h^{b}\right)}_{i}+c\right)$$

while for real-valued visible units, the conditional probability is defined as:

(6) $$P\left(v_{i}|h\right)=\text{normal}\left(\sum_{b}{\left(B^{b}*h^{b}\right)}_{i}+c,1\right)$$

The objective function to derive bases *B* and biases *d* and *c*, when provided with *L* training examples is defined as:

(7) $$\underset{B,d,c}{\text{min}}-\sum_{l=1}^{L}\text{log}\sum_{h}P\left(v^{\left(l\right)},h^{\left(l\right)}\right)+\lambda \sum_{b=1}^{n_{b}}{\left(s-\frac{1}{L}\sum_{l=1}^{L}E\left[h_{b}^{\left(l\right)}|v^{\left(l\right)}\right]\right)}^{2}$$

The first term denotes the negative log likelihood of the input data. The second term denotes the regularization term, with λ being the regularization constant. The sparsity constant *s* ensures that the hidden units have sparse activations resulting in more interpretable features. Given that computing precise gradients for the likelihood term is computationally expensive [18], contrastive divergence was employed to train the CRBM [30].

**Fig. 1.**
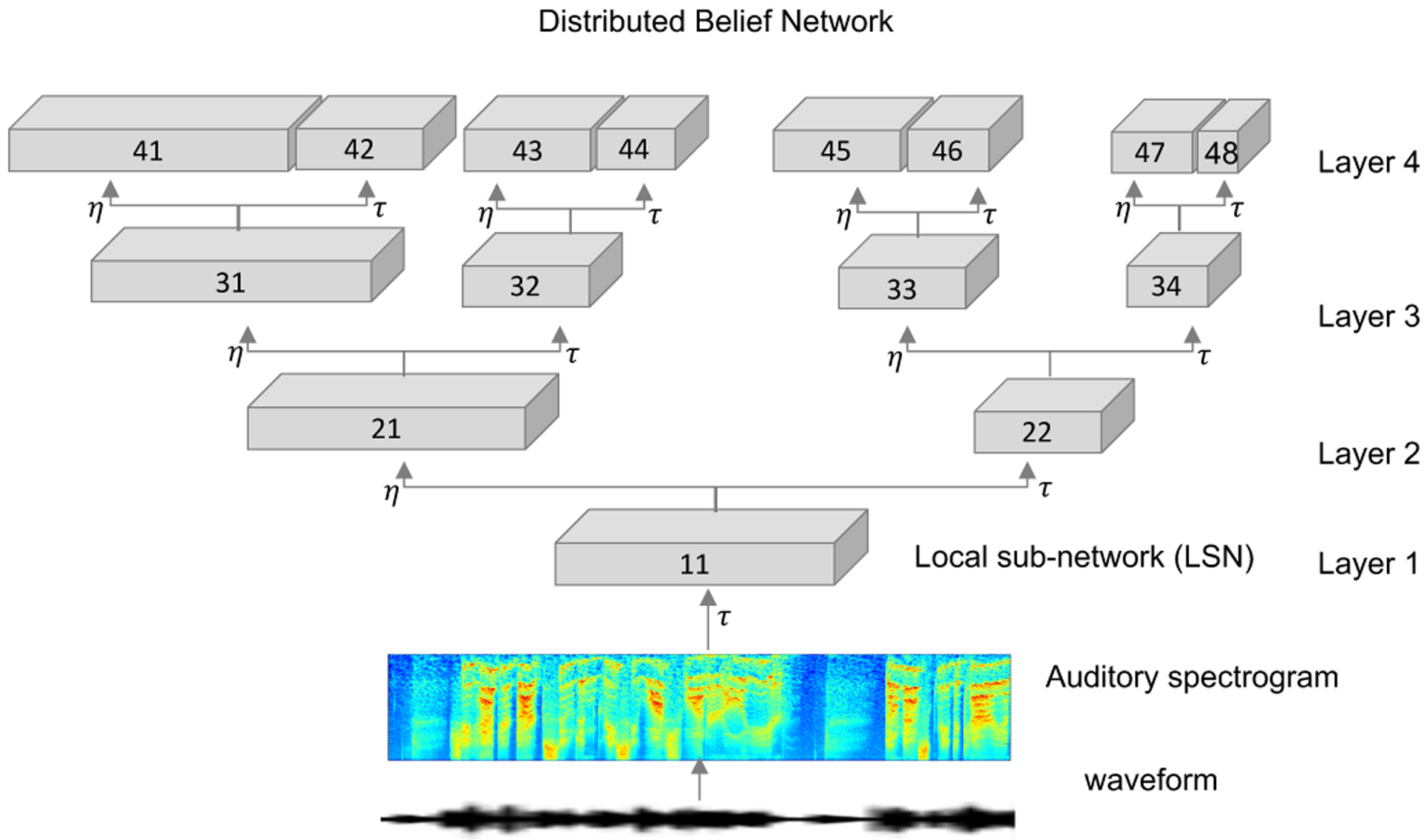
Distributed belief network with auditory spectrogram. Each block represents a LSN with CRBM hidden units. *η* and *τ* denote pooling ratios with *η < τ*. The numbers γζ within each of the boxes serve as sub-network identifier, signifying layer γ of the DBN and sub-network ζ within the layer. The sub-network numbers increase from left to the right within each layer.

In the current work, we build on this basic architecture and explore a distributed space to span a wide range of bases functions that capture the natural variability in everyday sounds. Instead of a standard multi-layer hierarchical network, the proposed architecture is a distributed belief network (DBN) as shown in figure 1, inspired from such distributed representations reported in auditory cortical networks [9]. This setup takes as input an auditory spectrogram and each local sub-networks (LSN) in the tree-like structure represents a latent representation with CRBM units. The numbers γζ within each of the boxes serve as sub-network identifier, signifying layer γ of the DBN and sub-network ζ within the layer. The sub-network numbers increase from left to the right within each layer. CRBM units are Gaussian-Bernoulli units in the first layer (with auditory spectrogram as input) and Bernoulli-Bernoulli units in rest of the layers. Left branches of the tree structure represent hidden layers estimated with a probabilistic pooling ratio of *η*, while right branches indicate probabilistic pooling by a ratio of *τ* along time axis, with *η < τ*. The frequency axis is faithfully translated across layers without any manipulations. As outlined next, this scheme is trained in an unsupervised fashion using a wide-range of natural sounds in order to capture inherent spectrotemporal dynamics in everyday sounds.

The DBN architecture developed in this work explores a number of propositions: (i) The hierarchical distributed setup estimates bases or spectrotemporal patches that encode spectrotemporal modulation features at varying abstractions and temporal rates, similar to tuning characteristics of the cortical neurons in the biological system [31, 32]; (ii) The hierarchical flow of the DBN results in increasing abstractions of the incoming signal making it suitable for representing sound classes of varying complexity and variability which is ideal for acoustic object classification; (iii) The redundant nature of the distributed network provides complementary information about spectrotemporal modulations in an incoming signal allowing a more integral mapping of audio signals representation where each LSN contributes from its own vantage point. These points will be explored in the analyses that follow.

## III. Distributed object classifier

Building on this distributed DBN representation, we explore its benefit for acoustic object classification in everyday acoustic scenes. We hypothesize that individual LSNs will capture different aspects of acoustic events that populate a scene; with the DBN —as a whole— faithfully encoding a more complete picture of the acoustic scene.

**Fig. 2.**
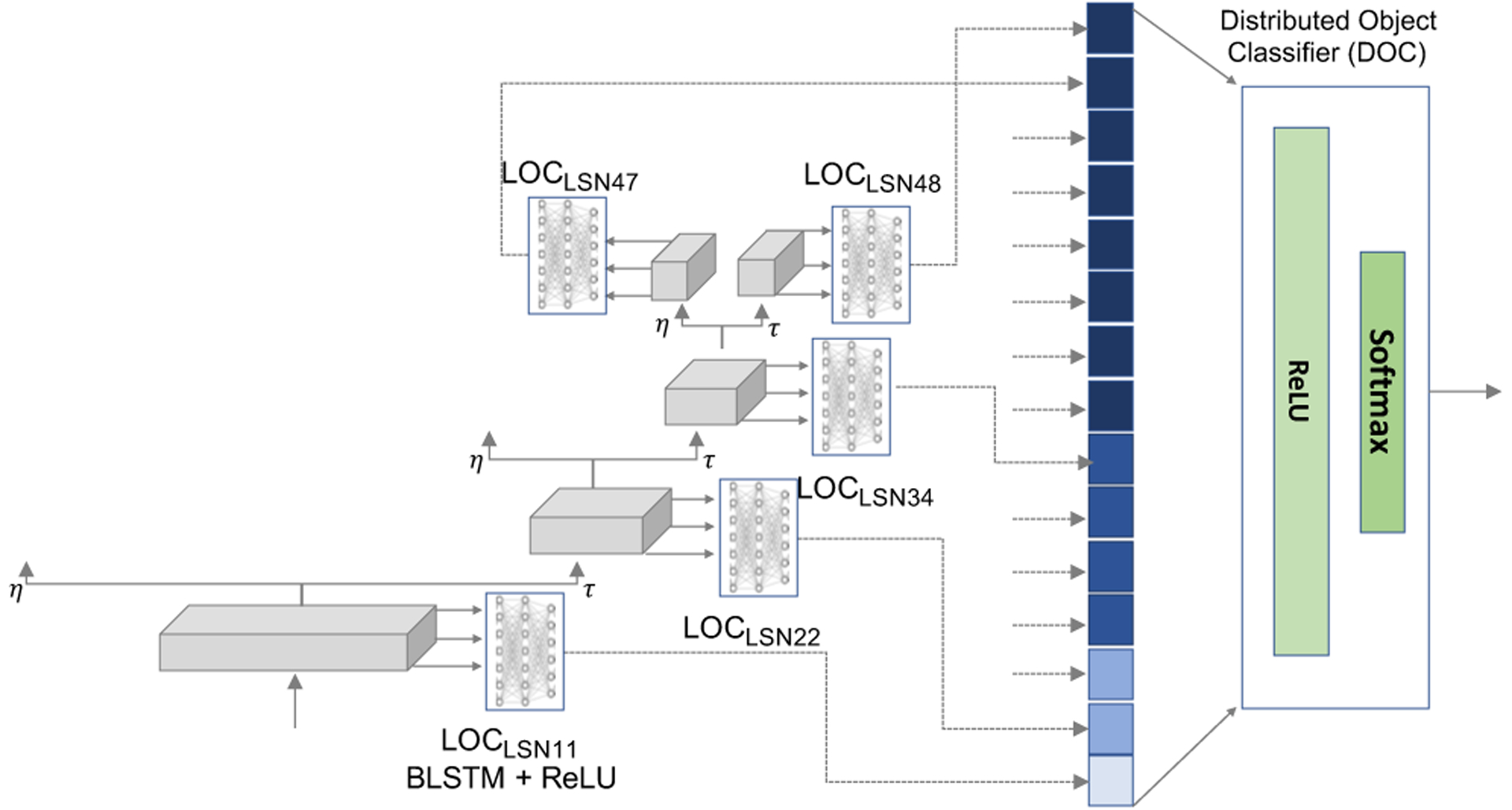
Acoustic object classifier. LOC is the local sub-network object classifiers and DOC is distributed object classifier. LSNγζ denotes layer γ sub-network ζ , with numbers increasing from left to right within a layer.

To develop an object classification system, we first train individual local object classifiers (LOCs) based on the activations from the respective local sub-network of the DBN for supervised classification of environmental sounds from the UrbanSound database. It should be noted that the CRBMs of the DBN are kept fixed and not re-tuned with the UrbanSound database. For instance *LOC*_*LSN*11_ in figure 2 refers to the local object classifier trained on the activations of the CRBMs in the local sub-network *LSN*11. Activations of these local classifiers are then used to inform a global distributed object classifier (DOC) trained to fuse local information across LSNs, as depicted in figure 2. The training is done in a sequential manner with the local object classifiers trained first, based on the outputs of which the distributed object classifier is trained. For local object classifiers , we employ a BLSTM (Bidirectional Long Short Term Memory) neural network followed by a dense rectified linear unit (ReLU) layer and a softmax layer. The global classifier DOC concatenates activations of LOC as features and uses a ReLU layer followed by a softmax operation to perform classification.

We hypothesize that the distributed nature of acoustic analysis will be beneficial especially in mismatched noisy conditions. In the presence of maskers, different LSNs of the DBN will capture both the acoustic object of interest and the masker from different vantage points in terms of spectrotemporal resolution and hence can maintain high fidelity representation of objects of interest. This will enable the DOC which is based on multiple redundant views of the clean acoustic object, to recognize acoustic objects at a higher accuracy than a traditional deep neural network.

## IV. Attentional feedback from local memory

Building on this setup, we further explore benefits of adaptive read-outs guided by feedback from prior knowledge. We are specifically interested in feedback from selective attention that can further enhance representation of acoustic objects of interest and suppress any maskers or competing sound sources. Mechanisms of endogenous attention have been shown in brain networks to reshape the mapping of sounds of interest in order to facilitate their encoding in presence of other distractors [17, 33–35]. In order to effectively model this attentional feedback in conjunction with the distribution representation of the proposed DBN, we extend the DBN computational scheme with two processes, *local memory* and *attentional feedback*.

### A. Local Memory

**Fig. 3.**
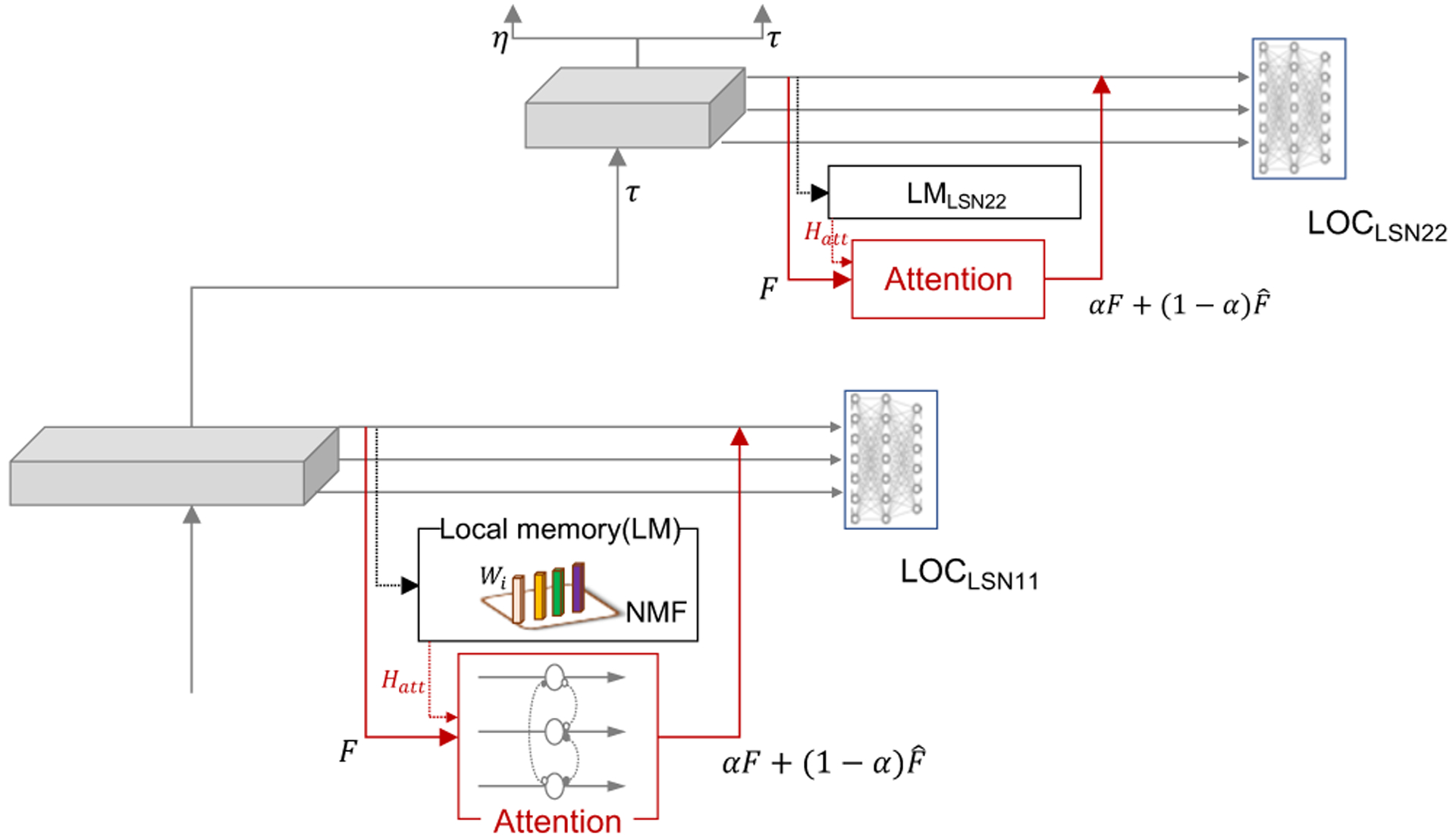
Schema for incorporating attention as a feature selector modulating the readout of the DBN. LM denotes local memory which are collection of NMF basis representing acoustic objects of interest. The attention block uses the feedback (*H*_*att*_) generated by the LM to modulate the respective local sub-network of the DBN.

We propose estimating a memory of each of the acoustic objects at every LSN, serving as a local memory of the target object from a particular vantage point. This local memory serves as prior knowledge of the object which is then used to modulate the belief network in a manner that enhances detectability of this target. In this work, we employ non-negative matrix factorization (NMF) [36] to model the local memory (LM) of each acoustic object of interest, represented locally at each LSN. At each of the 15 LSNs of the DBN, we estimate 10 NMF basis representing the local memory of the 10 classes of the UrbanSound database. The training procedure is performed as follows: when presented with multiple instances of an acoustic object belonging to a class, the hidden unit firing patterns along the time axis are extracted. These patterns are concatenated and used to estimate a single sparse NMF basis to represent the local memory of the object at a particular LSN. This collection of single NMF bases representing each of the acoustic objects at each of the LSNs is referred to as ‘local memory’ as shown in figure 3.

### B. Attentional feedback

During selective attention towards one of the acoustic objects on interest, local memories are employed to generate feedback in a manner that allows of enhancement of the representation of the object of interest while not creating false alarms if the object of interest is not present. These processes should operate in the conjunction with the local LOC and global DOC to enhance object classification. We tackle these adaptive additions to the network by leveraging the biological concept of temporal coherence [37, 38]. The principle of temporal coherence states that when attention is directed towards a particular feature of an acoustic object, all other features temporally coherent to the temporal activation of the anchor feature become bound together such that the acoustic object of interest stands out in the presence of masking acoustic objects. Thus, during inference, we use the local memory of the object of interest as anchoring feature and determine its activation pattern. All the hidden units of that LSN with an activation pattern temporally coherent with the local memory are deemed to represent the object of interest and are emphasized, while the rest are suppressed. This modulation can be interpreted as attention acting as a feature selector where the readout from the sensory mapping process is modulated to aid behavior. Effectively, the attentional feedback operates by modulating the latent information captured by each of the LSNs at the inference stage such that the object being attended to is enhanced while suppressing the maskers.

Specifically, local memory is applied as attentional feedback during inference as follows:

(8) $$F\approx \left[W_{att}W_{2}\ldots W_{M}\right]\left[\begin{array}{c} H_{att}\\ H_{2}\\ \vdots \\ H_{M} \end{array}\right]$$

where $F\in {\mathbb{R}}^{B\times N}$ represents the firing pattern of the *B* hidden units of a local sub-network over *N* frames, when presented with a multi-object acoustic signal. The firing matrix *F* is next factorized along *M* dimensions. During factorization, we incorporate attention by keeping the first basis of the decomposition matrix fixed as *W*_*att*_, which is the local memory of the object towards which attention is being directed. The first row of the mapped activation matrix, *H*_*att*_, represents the activation pattern of the local memory; while remaining rows capture any other objects present in the audio input. As shown in figure 3, *H*_*att*_ serves as the feedback generated by the local memory, which is then utilized by the attention block to modulate the encoded features.

In the attention block, correlation of the firing patterns of each of the units of the *F* matrix with *H*_*att*_ is estimated as shown below. *f*[*i*] is the correlation of the *i*^*th*^ unit with the activation pattern of the local memory:

(9) $$f[i]=\sum_{n=1}^{N}F[i,n]*H_{att}[n]$$

Using principle of temporal coherence, units deemed incoherent with the attended object (below a threshold *β*) are set to zero; while units that are above the threshold are retained, as outlined in the following equation:

(10) $$\hat{F}[i,n]=\{\begin{array}{ll} 0, & \;\text{for}\;f[i]<\beta \\ F[i,n], & \;\text{for}\;f[i]\geq \beta \end{array}\;\forall n\in 1,\ldots ,N$$

Finally, a weighted sum is used to modify the final activations of the LSN, as described below:

(11) $$\alpha F+(1-\alpha )\hat{F},\;0\leq \alpha \leq 1$$

These modified activations (figure 3) are then propagated to the higher layers of the DBN and the local sub-network object classifiers.

## V. Results

### A. System setup and parameters

The core DBN architecture was trained using 3 hours of speech from the TIMIT database [39], 4 hours of BBC Sound effects database [40] and 2 hours of instrumental solo music used in [41], all sampled at 16*kHz*. Training on these databases allows us to derive a feed forward sensory mapping system that can faithfully span the modulation space occupied by a large variety naturally occurring sounds in a task agnostic manner. Inputs were auditory spectrograms with 128 frequency channels spanning 5.3 octave and a temporal resolution of 6*ms* per frame. The LSN in the first layer *LSN*11 consists of 300 Gaussian-binary CRBM units with basis of dimensions 128 *×* 6. Each of the remaining 14 LSNs across the layers two, three and four of the DBN consisted of 300 Gaussian-Gaussian CRBM units each with bases of dimensions 300 × 6. The pooling parameters were fixed to *η* = 1 on the left branch and *τ* = 3 on the right branch following the schema shown in figure 1. Therefore, the *fastest* LSNs operates at a resolution of 18*ms* while the *slowest* local network (*LSN*48) operates at the rate of 486*ms*. The DBN was trained layer by layer using contrastive divergence to train the CRBMs in each layer, with a sparsity constant *s* = 0.05 and regularization constant λ = 5 (equation 7).

The object classification component was trained in two steps: Each of the local sub-network object classifiers (LOCs) were first trained using a neural network architecture consisting of 50 BLSTM units in each layer, followed by a fully connected layer with 50 ReLU units and a softmax layer. Next, the fully connected layer activations of each LOC were used as input with dimensions 750 (15 LSNs and 50 units in each LOC) to a global network with a single layer with 50 ReLU units and a softmax layer. For training LOCs and DOCs, we employed Adam [42] with a constant learning rate of 0.001, with a epoch size of 50. *l*2 regularization was used with a penalty value of 0.001.

The local memory component was estimated using a random subset of data from each object class in the training set from the UrbanSound database. A single NMF basis was estimated at each LSN for each object class using Frobenius norm measure [36]. For the attentional feedback, the threshold parameter *β* was set empirically to −0.1 (Eq. 10), while *α* which represents the weighting between the features encoded by the DBN before and after attention was set at 0.7 (Eq. 11).

#### Baseline system comparison:

A five layer convolutional neural network (CNN) was employed as baseline system, following the implementation proposed by the authors of the UrbanSound database in [43]. The UrbanSound database consists of environmental sounds from following classes, air conditioner, car horn, children playing, dog barking, drill, engine idling, gunshot, jackhammer, siren and street music.

#### System testing and validation:

The system was validated using ten fold cross-validation using the splits prescribed by the authors of the database. Local memory was estimated de novo for each validation round. Given the varying signal durations, system testing was performed by extracting random contiguous three seconds from each sound sample, following the procedure proposed in [43]. The system was always trained with clean data and tested in matched clean conditions, as well as noisy conditions, generated by adding competing sounds from one of three sources: 1) signals from another class from the UrbanSound dataset used as maskers; 2) sounds from the NoiseX database [44]; 3) sounds from Rouen auditory scene database [45] which consists of sounds recorded from natural auditory scenes such as a tube station, a student hall and a market. The initial system performance was evaluated without the attentional feedback. Incorporating this feedback was done in a separate stage to assess its benefit in modulating the system’s output depending on the sound class of interest.

### B. Latent Modulation span of the DBN

**Fig. 4.**
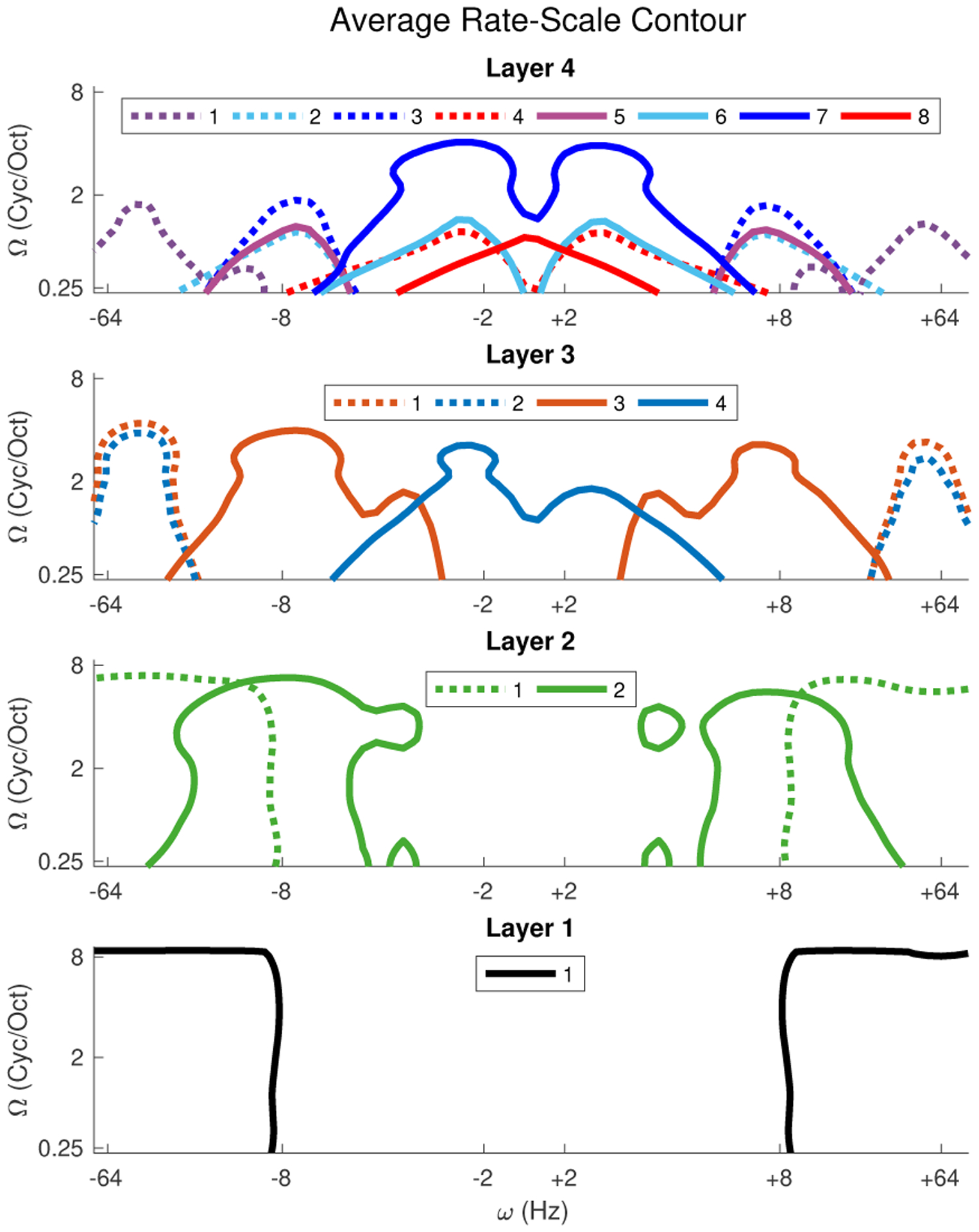
Each contour represents the average rate-scale spanned by the basis of the particular LSN. Faster rates and a broad range of scales captured by LSNs of the lower layers (rows 4 and 3). LSNs of the higher layers capture slower scales and slow temporal rates less than 4*Hz* captured by sub-networks such as *LSN*34 and *LSN*48.

First, we analyze the effectiveness of the proposed deep belief architecture in capturing the spectrotemporal modulation space of natural sounds in a distributed fashion. The basis functions at each LSN are reconstructed approximately as a linear combination of the bases from the lower layer. As stated in our main hypothesis, these bases are viewed as decompositions of the auditory spectrogram from different vantage points. Each of these functions is convolved in time and frequency with an array of 2-dimensional Gabor filters spanning temporal modulations (or rates) *±*2 − 64*Hz* and spectral modulations (or scales) 0.25 – 8 cycles/octave to estimate the the average rate-scale spread at each LSNs. Figure 4 shows the contour plot of the average rate-scale spread of bases at each local sub-network (LSN). These profiles highlight that LSNs in the first two layers span the entire range of scales while capturing faster temporal rates. As the signal propagates to higher layers, slower (i.e. more abstract) scales are asserted along with slower temporal rates, likely enunciated by the pooled LSNs on the right branch of the tree like structure. As hypothesized, the DBN spans the entire spectrotemporal modulation space in natural sounds [4], but tiles the space in distributed albeit redundant fashion.

**Fig. 5.**
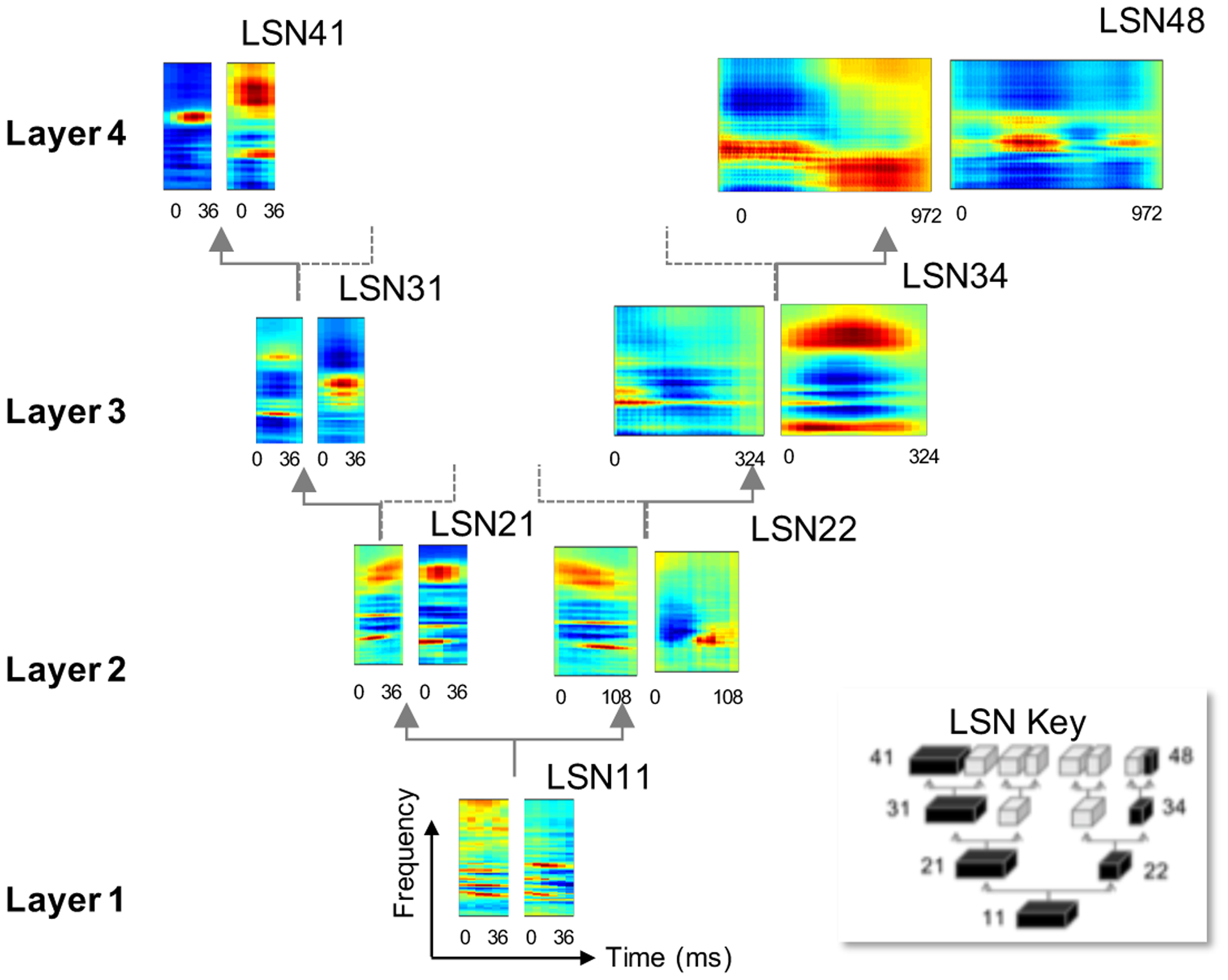
Example basis from 7 local sub-networks. The LSN key illustrates the LSNs from which example basis have been illustrated. The basis are spectrotemporal filters with red color indicating activation and blue color inhibition; with frequency axis spanning 8*kHz* and the time axis is indicated in milliseconds - It should be noted that the images are not to scale.

Taking a closer look at *individual* basis functions, their profiles reflect highly-structured selectivity along time and frequency axes that is reminiscent of similar patterns reported in cortical neurons [46–48]. Figure 5 shows example functions derived at different nodes of the DBN network. The figure contrasts examples from the leftmost and rightmost LSNs at each layer (as indicated by the LSNs in black in the figure key). Focusing on the left branch, all bases from *LSN11, LSN21, LSN31* and *LSN41* operate at the same rate and span 36*ms*. The examples show a greater degree of abstraction as the signal propagates through the hierarchy with *LSN11* capturing more detailed spectrotemporal patterns with larger activation regions, while the higher nodes appear sparser and broader in coverage. In contrast, the rightmost branch spans increasingly greater temporal profiles with *LSN22, LSN34* and *LSN48* covering 108*ms*, 324*ms* and 972*ms* respectively. Propagation through the hierarchy along this branch also reveals increasing abstraction across slower temporal dynamics highlighting events ranging from tens of milliseconds to hundreds of milliseconds.

### C. System performance in matched conditions

**Fig. 6.**
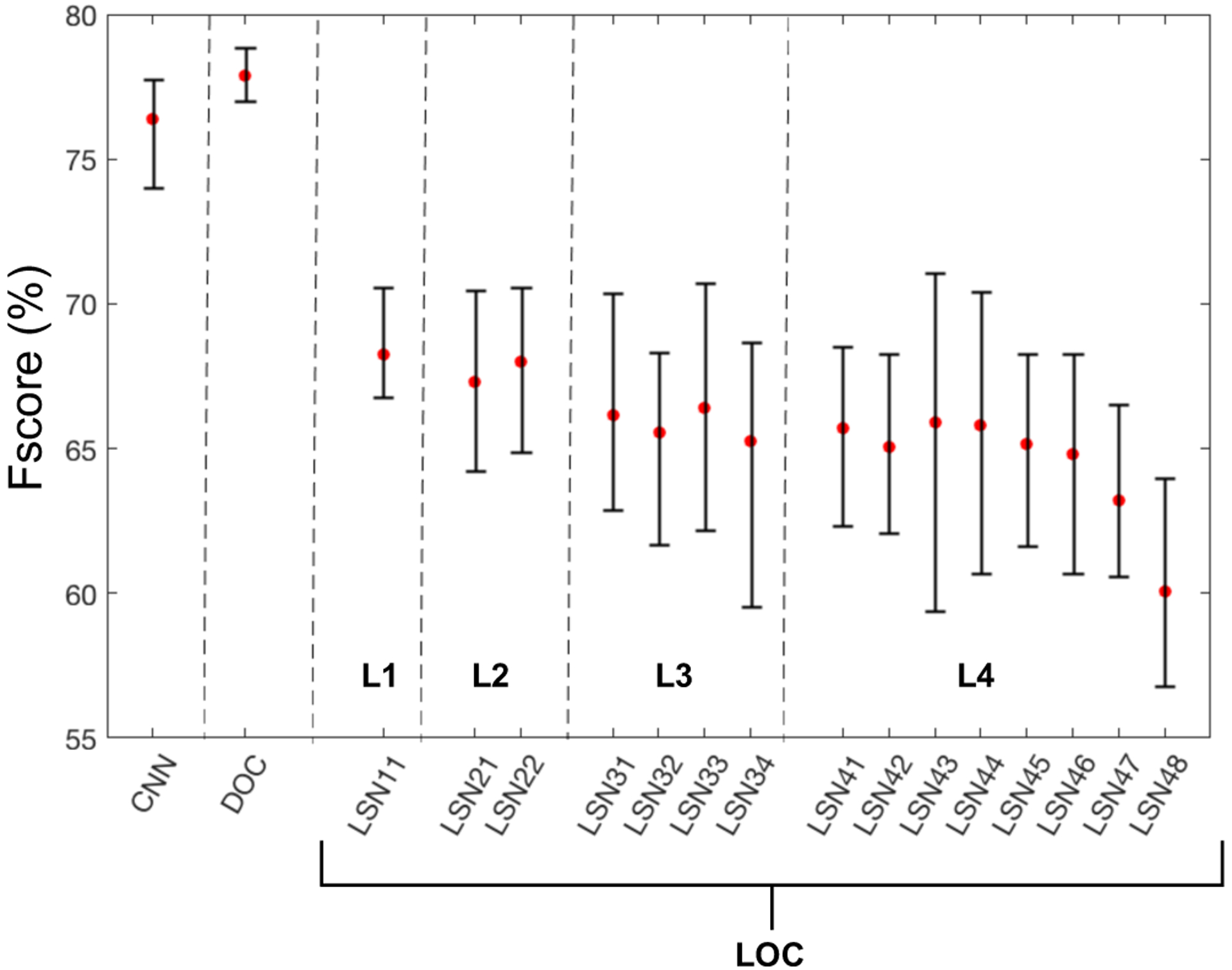
Performance in terms of the F-score. LOC indicates local sub-network object classifiers with the errorbar representing the spread across 10 fold cross-validations.

Figure 6 shows the performance of the global system (DOC) as it compares to the CNN baseline; as well as the performance of the individual distributed LOCs in matched clean conditions. Labels for LSN_γζ_ in figure 6 follow the same structure outlined earlier with γ indicating layer and ζ indicating the sub-network number from left to right. The performance results show that the proposed system with an average F-score of 77.8% performs marginally better than the CNN baseline with an F-score of 76.7%. Furthermore, individual LOCs only achieve in the range of 60 – 68%, though, interestingly, LSNs in lower layers appear to perform marginally better. This result is not surprising given that sounds in the UrbanSound database are more dominated by sharper sounds that are well characterized by faster rates (e.g: jack hammer, gun shot and air conditioner). There is also a likely contribution of the powerful BLSTM that takes advantage of the detailed mapping in the lower layers in order to better capture the discriminatory information, especially in matched conditions, in contrast to increasing abstractions at higher layers. It should be noted again that the DBN, which serves as a feature extractor for classifier was not trained using the UrbanSound database.

### D. Performance in mismatched conditions

**Fig. 7.**
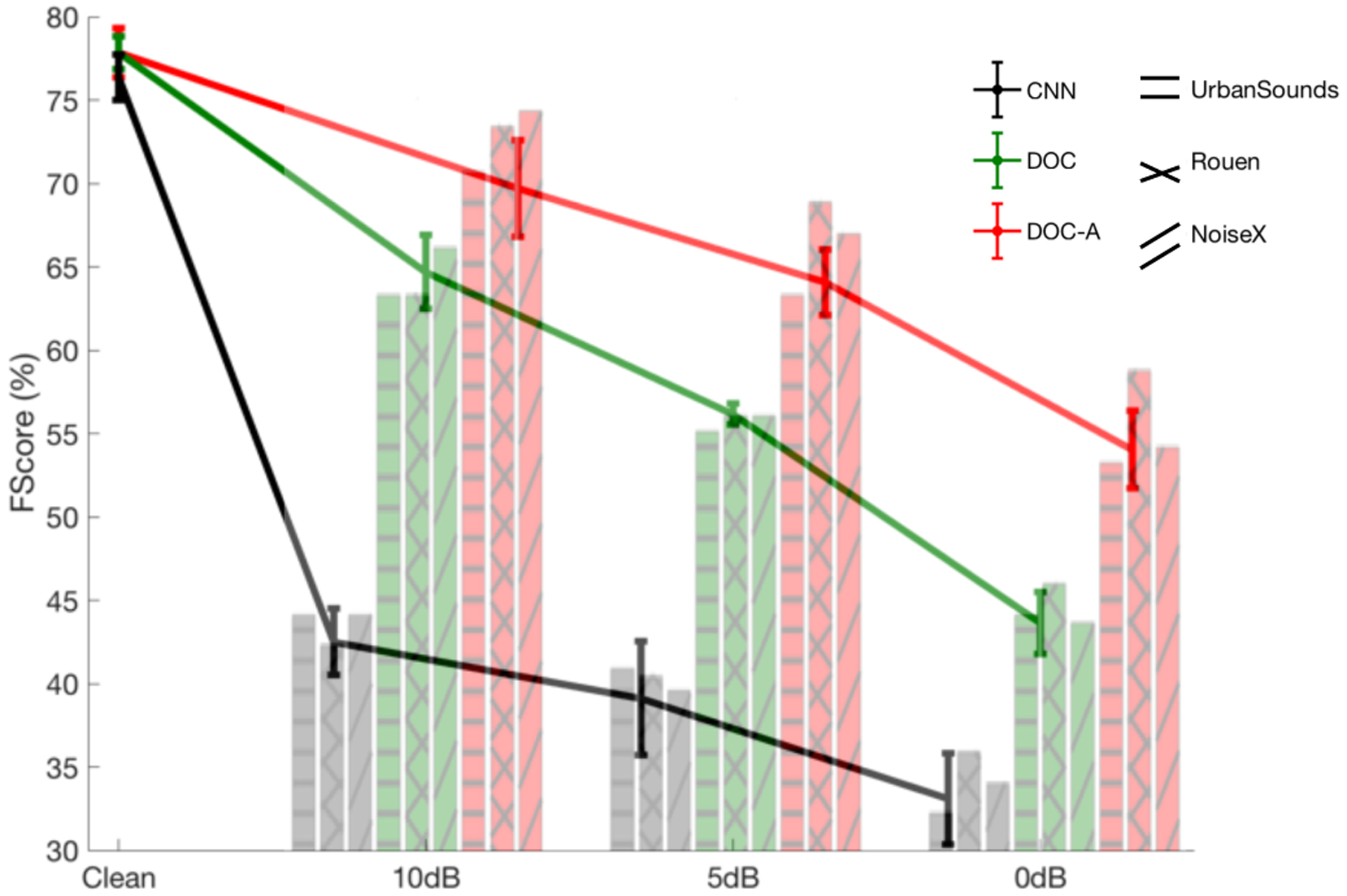
Performance in clean matched conditions and average F-score across 3 noisy mismatched conditions. The bar chart in the background shows the average performance in each of the noise databases used in this study. The errorbar shows the average F-score and spread across the 3 noisy conditions.

We hypothesized that the distributed nature of the feature extraction and the object classification system will be beneficial in noisy conditions. To test this hypothesis, our analyses explores three types of distractors (competing scenes from the same database, non-stationary noise sources or distractors from everyday scene from another database). Figure 7 shows the performance of the system at three signal to noise ratios (SNR). The errorbar shows the average F-score and spread across the 3 distractor types, while the bar plot in the background shows the average F-score for each of the mismatched conditions. The efficacy of the proposed system clearly stands out in mismatched conditions, with the DOC classification system (green curve) performing significantly better than the CNN system. The relative improvement is 31.4% at the 0dB SNR. It should be noted that like the baseline CNN, the DOC is trained only in clean conditions.

**Fig. 8.**
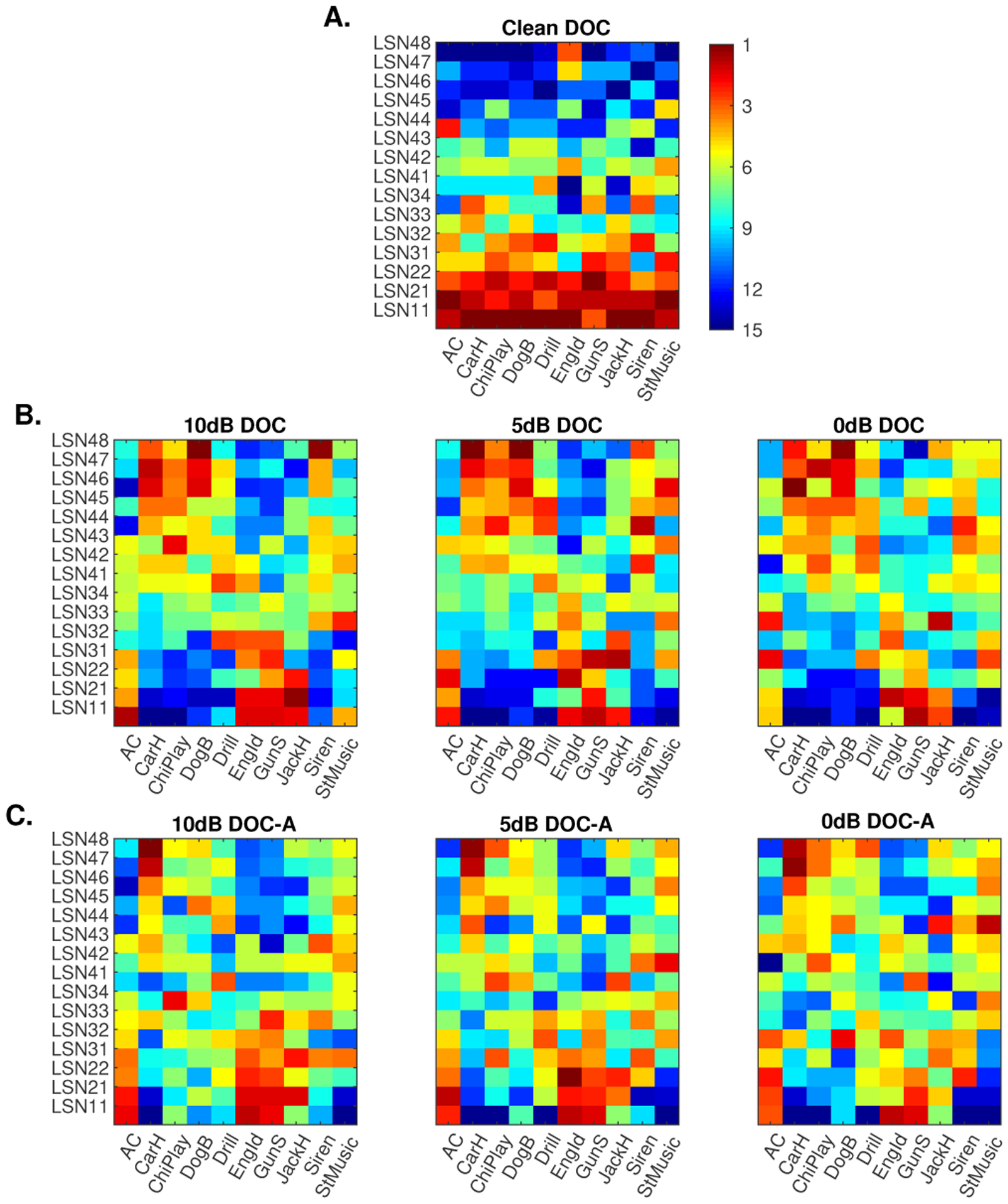
Ranking of the LSNs in terms of performance for each of the classes. Row A shows the ranking in the clean conditions. Row B shows average ranking across the 3 additive noise conditions without attentional mechanisms. Row C shows average ranking across the 3 additive noise conditions with attention. Given that there are 15 LSNs in the DBN, the average ranking of the individual LSNs ranges from 1 to 15, with more red indicating better performing LSNs and blue indicating the lower ranked LSNs.

In order to gain better insight into the benefits of the distributed scheme, we examine the contribution of individual local LSN network in the final classification performance (in terms of F-score) by ranking them from best performing to worst (from 1 to 15). Figure 8A shows the ranked contribution of each LSN in recognizing individual classes in the dataset in the clean condition. As seen earlier in figure 6, lower faster LSNs contribute the most to the overall performance of the system, consistently across all sound classes.

Figure 8B depicts the average ranking of each LSN in noisy conditions averaged across all distractors. The figure shows the disruption of the LSN ranking as a function of SNR where we notably observe stronger contributions of higher LSN nodes, especially for certain auditory objects, as well as a more spread out contribution across all layers. We specifically note how slower LSNs in the higher layers seem to fare better for few classes such as *car horn*, *children playing* and *dog bark*.

**Fig. 9.**
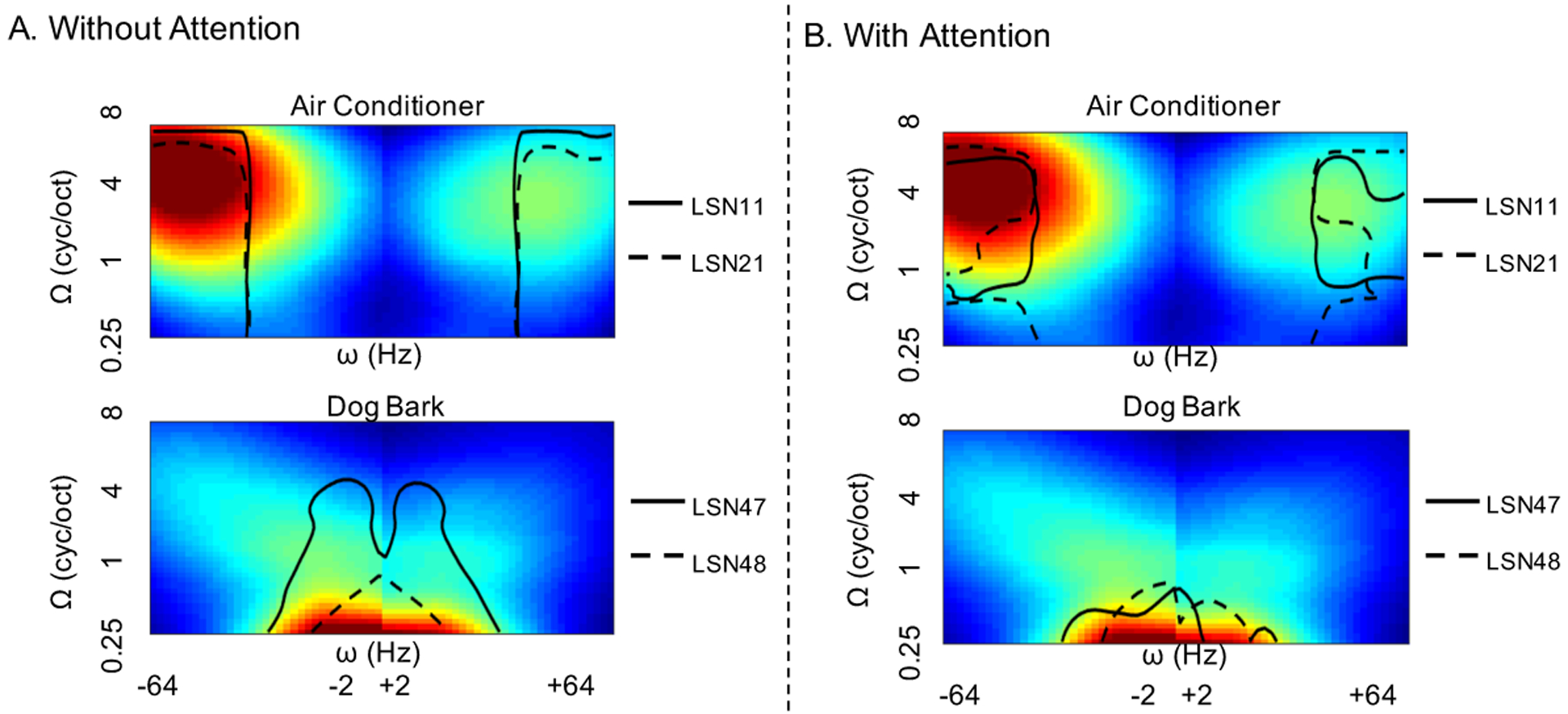
Average rate-scale spread for 2 classes from the UrbanSound database, Air conditioner and Dog bark. Red color signifies high energy and bluer regions low energy. The black lines denote the contour lines of the average rate-scale spread of the LSNs indicated. In block A, the contours lines indicate the rate-scales captured by the LSNs mentioned without attentional bias. In block B, the contour lines indicate the rate-scales captured by the LSNs mentioned with attentional bias. The rate-scale estimate of each of the basis at a particular LSN is multiplied by the NMF weights of the local memory of the acoustic object object before estimating the contour.

Next, we take a close look at spectral and temporal dynamics of sounds in each class in the database and examine how the contribution of individual LSNs contributes to the global performance of the system, especially in noise. Figure 9A shows the average rate-scale spread of 2 classes *Air conditioner* and *Dog bark*. In this case of the *Air conditioner* class, it can be seen that it is dominated by faster scales and rates, modulations primarily captured by the LSNs of the lower layers. This is illustrated by the juxtaposed contour lines depicting the average rate-scale spread of basis from *LSN11* and *LSN21* (depicted earlier in figure 4). Therefore across matched and mismatched conditions the LSNs in the lower layers perform best, as can be seen from the LSN ranking in figure 8. Whereas in the case of *Dog bark* class, slower modulations dominate and hence it benefits majorly from views captured by the slower LSNs in the 4th layer such as *LSN*47 and *LSN*48, as indicated by the contour lines and LSN rankings in noisy conditions.

### E. Performance with attentional mechanisms

Finally, we examine the contribution of attentional feedback and local memory in further improving classification performance. As outlined earlier, feedback is deployed if the system is attentive to an object of interest (e.g. siren) and is actively engaged to detect this target of interest (while balancing false alarms). The red curve in figure 7, denoted as DOC-A shows the system performance when attentional feedback is deployed to modulate the readout of each LSNs of the network. As expected, attentional feedback leads to further improvements to the overall classification performance with notable advantages in noisy conditions. The relative improvement is 24.4% at the 0dB SNR relative to the DOC without attention and 54% relative to the CNN baseline system. Figure 8C looks at the contribution of individual LSNs in the overall performance with attentional feedback in terms of ranking. In this case too, we can see that the contribution of LSNs increases higher up the hierarchy, in terms of average ranking in mismatched conditions, especially in the lower SNR conditions.

The contour lines in black in figure 9B further illustrate the manifestations of using local memory to modulate the network. In the case of the *Air conditioner* class, the rate-scale estimate of each of the basis of sub-network *LSN*11 and *LSN*21 was multiplied by the NMF weights of the local memory of the *Air conditioner* object from the respective LSN, before estimating the contour for the LSN. Thus, the contour lines signify the average rate-scale spread of the LSNs under attentional feedback from the local memory of the object being attended to. As can be seen, with attentional bias, both sub-networks *LSN*11 and *LSN*21 capture a sparser region of the modulation space (in comparison to the contour lines in figure 9A); highlighting regions of the rate-scale modulation space where the *Air conditioner* acoustic object dominates. Similar behavior can be seen for the *Dog bark* case too, where the contour lines in figure 9B, shrink in comparison to 9A, with focus on the very slow rates and scales.

## VI. Discussion

The current study explores a distributed scheme for encoding acoustic characteristics of natural sounds. Inspired by a bio-mimetic architecture in the human auditory cortex [9], the proposed model explores a novel generative distributed belief network which spans the spectrotemporal modulation space occupied by everyday soundscapes in a hierarchical and multi-resolution tiling. This framework is trained on independent datasets to ‘learn’ a distributed set of complex sound profiles comprising spectral and temporal characteristics, allowing a supervised classification system to leverage these multiple mappings to yield robust acoustic object classification of an UrbanSound database. This scheme not only achieves on par performance with a state-of-the-art convolutional neural network framework in clean conditions, but largely outperforms this baseline in mismatched conditions, where sounds of interest are present in the midst of competing distractors. In addition, we incorporate mechanisms of attentional feedback that allows the belief network to deploy local memories of sounds targets stored at multiple vantage points to bias the activations of the network, hence resulting in further improvement of object classification in unseen noise conditions.

This work can be interpreted in the context of recent efforts that have sought to address the problem unseen noise conditions using generative neural networks [49–51]. In [49, 50], variational autoencoders (VAE) are used to learn latent representation for speech in an unsupervised manner. It is shown that during inference, by performing latent space arithmetic operations, information not pertinent to speech recognition can be suppressed in unseen noise conditions leading to improved robust speech recognition. In [51], an integrated VAE and NMF based framework is proposed for speech enhancement in unseen noisy conditions. In this case, the VAE is trained to map clean speech onto the latent space. In unseen noisy conditions, speech is enhanced by using the VAE to first generate a prior estimation of the clean speech by decoding from the latent space, while explaining away the low ranked noise using NMF. Across these frameworks, the broad idea is to first learn a generic latent space to encode the acoustic signal in clean conditions. During inference, the latent space is modulated in novel noisy conditions in a semi-supervised manner to match prior estimated statistics so as to ensure improved decoding from the latent space.

Viewed in this context, the proposed belief framework expands on these concepts by leveraging a bio-mimetic hierarchical formulation. The generative DBN architecture spans the spectrotemporal modulation space and maps the acoustic signal onto a distributed latent space instead of a single latent space representation. This allows for a more decentralized representation of the auditory scene that proves particularly useful in mismatched conditions. Different LSNs of the system, which are essentially latent spaces representing the auditory scene from different vantage points, capture the objects of interest with varying fidelity. Therefore, the deterioration in performance of the distributed object classifier trained on the distributed latent space representation is considerably lesser when compared to the CNNs in mismatched conditions. Further, the attentional mechanisms implemented in this work can be viewed as modulating the latent space as proposed in [49–51] but in a more distributed manner. The notion of distributed local memory allows attentional mechanisms to modulate the representation captured within the purview of each LSN.

The proposed framework also offers interesting avenues to explore as future work. In this study, the inference process pools information across all the LSNs of DBN to be utilized by the distributed object classifier, irrespective of the fidelity of the encoding captured by the LSN itself. This concept can be modified to adjust the contribution in the integration stage by allowing maximally informative LSNs to further inform the classification stage, akin to processes of stream selection, often employed in multistream frameworks for automatic speech recognition [52, 53]. Furthermore, the proposed scheme modeled attention as a feature selector, that is attentional mechanisms modulate the latent representation which in turn are used as features by the BLSTM based neural networks. Attention can also be modeled as adapting the very basis of the DBN that encode the sensory cues leading to faster inference on providing more stimulus from similar conditions. This kind of the attention driven adaptation has been widely observed in the cortical regions [11–13], and successfully modeled computationally using both linear and nonlinear transformations of the mapping stage [54, 55]. The deployment of attentional feedback in the current setup could be re-interpreted as process that re-tunes the basis functions of the DBN such that encoding of incoming signals highlights relevant sensory cues regardless of presence of competing distractors in the input signal. Such implementation would have interesting implications for tasks such as robust speech enhancement.

Finally, while the use of the generative model here has enabled us to illustrate the usefulness of distributed mapping and attentional mechanisms for the task of classification of environmental sound classification, the proposed generative framework in its current form might be limited in its ability to match the state of the art deep networks based speech enhancement or source separation systems. Reconstructing the stimulus back in the spectrotemporal space from the latent representations of the DBN is not straightforward. Further, the use of NMF as a standalone block to implement attentional mechanisms can be computationally expensive. A future goal is to integrate the distributed representation and attention mechanisms based on distributed memory of acoustic objects within a deep neural network that is trainable in an end-to-end manner for tasks such as speech enhancement and source separation.
